# Transformation of the private healthcare sector in the Republic of Kazakhstan following healthcare reforms

**DOI:** 10.3389/fpubh.2025.1608557

**Published:** 2025-09-01

**Authors:** Smagulov Alibek, Kurakbayev Kuralbay, Baimakhanov Abylai, Zhandossov Olzhas, Abilkaiyr Nazerke, Zhakupova Maiya, Bapayeva Magripa, Kapanova Gulnara, Alikeyeva Galiya, Makhanbetkulova Dinara

**Affiliations:** ^1^Department of Population Health and Social Sciences, Kazakhstan Medical University “KSPH”, Almaty, Kazakhstan; ^2^Department of Health Economics and Insurance Medicine, Kazakhstan Medical University “KSPH”, Almaty, Kazakhstan; ^3^Faculty of Postgraduate Education, Kazakh National Medical University named after S.D. Asfendiyarov, Almaty, Kazakhstan; ^4^Department of Public Health, Kazakh National Medical University named after S.D. Asfendiyarov, Almaty, Kazakhstan; ^5^Kazakhstan Medical University "KSPH", Almaty, Kazakhstan; ^6^Department of Health Policy and Organization, Al Farabi Kazakh national University, Almaty, Kazakhstan; ^7^Department of Nursing, Kazakh National Medical University named after S.D. Asfendiyarov, Almaty, Kazakhstan

**Keywords:** private practice, government, public-private partnership, healthcare, Kazakhstan

## Abstract

**Background:**

In Kazakhstan, transformation of the private healthcare sector as the country transitioned from lower-middle-income to upper-middle-income status has rarely been a subject of academic debate. This study aimed to analyze of health sector indicators disaggregated by type of ownership over the period of 10 years (from 2011 to 2020).

**Methods:**

This was a retrospective cross-sectional study, which was based on official healthcare statistics presented by the Ministry of Health. Relative change (RC) was computed to identify trends in changes over time and was expressed as a proportion with 95% confidence intervals.

**Results:**

In contrast with the government-owned health facilities, the overall number of private facilities increased over time, although this growth was less obvious in per capita terms. The number and density of private PHC facilities grew more substantially than those of outpatient facilities. PHC practitioners employed by the government-owned facilities admit more patients than practitioners of the private PHC facilities do. There is an uneven presence of the private health sector in different medical specialties with maternity, ophthalmology, dentistry, narcology, multidisciplinary, as well as palliative and nursing care being the most common.

**Conclusion:**

Such data are needed by decision-makers to tailor public health strategies focused on the stewardship of the private health sector, which would help improve the availability and affordability of medical services.

## Introduction

The impact of the private health sector on the provision of healthcare services is difficult to underestimate as it has gained much popularity in different economies, including low- and middle-income countries (1). According to an operational definition of the World Health Organization (WHO), the term “private health sector” comprises both individuals and organizations who exist outside the public health sector and the direct control of the state and who provide healthcare services for philanthropic or commercial purposes (2). The private healthcare sector includes a broad range of providers—from traditional healers to specialized hospitals— while most healthcare systems globally combine both public and private services.

The Republic of Kazakhstan (hereafter–Kazakhstan) is a Central Asian state, which gained its independence in 1991 as a result of the Soviet Union’s breakdown. During the Soviet period, the country’s healthcare system is based on a model Semashko and thus, the private healthcare sector was largely non-existent (3). However, private health providers appeared in Kazakhstan already in the early 1990s and due to the public interest and the lack of state regulation, traditional medicine became very popular. When mandatory licensing and penalties for unlicensed medical activities were introduced (4), the private health market began to take shape. Currently, the country has a network of private health services, including hospitals, outpatient facilities, health centers, pharmacies, diagnostic centers and laboratories.

Kazakhstan has been continuously reforming its healthcare system from the early years of independence. Initially these reforms, were driven by the need to adapt to newly emerging challenges, but soon they became more systematic and comprehensive (5). The government adopted and implemented a series of national health plans directed at strengthening the public health system through various interventions. The “Salamatty Kazakhstan” (“Healthy Kazakhstan”) program for 2011–2015 set provisions on the development of the private healthcare sector and introduced the concept of public-private partnership. Specifically, the program provided for transfer of basic assets (facilities and equipment) from government to private health sector for lease and trust management, as well as award of public contracts to private healthcare providers (6). The “Densaulyk” (“Health”) program for 2016–2019 strengthened the state’s commitment for the further development of public-private partnership in healthcare sector. In particular, it was planned to increase the share of private healthcare providers to 41% by 2019 (7).

Despite ample evidence, the transformation of the private healthcare sector as the country transitioned from lower-middle-income to upper-middle-income status (8) has never been a matter of academic debate. Therefore, this study was aimed at the analysis of health sector indicators disaggregated by type of ownership (government vs. private) over the period of 10 years (from 2011 to 2020). Specifically, we focused on the density of outpatient and inpatient facilities both in the fields of primary healthcare (PHC) and specialized medical care, as well as on certain aspects of productivity, including patient visits and hospital admissions.

## Methods

### Study design and procedures

This study relies on official healthcare statistics presented by the Ministry of Health (MoH) in the form of a statistical yearbook “Public health in the Republic of Kazakhstan and activities of healthcare facilities.” This yearbook is an annual edition developed by the Republican Center for Electronic Health (RCEH) on the basis of reports submitted to the MoH (Bureau of National statistics. Statistical collections, 2011–2020). In Kazakhstan, it is a standard practice to obligate the heads of all healthcare facilities, irrespective of the type of ownership, to report to the provincial health authorities on annual activities using a special form. At the end of each year provincial health authorities submit these aggregated data to the MoH and RCEH prepares an annual report, which is divided into 20 subsections. For the purposes of this study, we extracted information contained in the “Health network,” “Health personnel,” and “Beds” subsections. The yearbook is issued since 1999 and all editions are available for free access (9). Information on the use of this statistical compilation in healthcare research has been presented elsewhere (10, 11).

In addition, we addressed to the annual edition of Demographic Yearbook issued by the Agency of Statistics of Kazakhstan to obtain the data on population size in the country and its provinces (12).

### Data presentation

The RCEH presents all data in absolute numbers for the whole country and separately for each province in dependence with the type of institutional appointment, i.e., MoH or other ministries. Information on the private health sector is presented separately. For our analysis, we combined health facilities under governance of MoH and other ministries into one category – the government health sector – and compared them with the private. All data were retrieved for the period of 10 years (from 2011 to 2020). While the majority of indicators cover the full 10-year period (2011–2020), data on specific out-patient and in-patient facility types were only available from 2013 onward and are thus reported for 8 years (2013–2020).

The “Health network” is a subsection of the statistical yearbook that presents disaggregated information by type of healthcare facility (outpatient vs. inpatient) and its specialization (primary healthcare vs. specialist care). As a first step, we examined the areas of specialization of government and private healthcare facilities and observed a significant under-representation of the private sector in many medical fields. To address this issue, we decided to extract data only for those types of specialist care in which at least three private facilities were operating during any year of the observation period. This threshold was applied to avoid bias and ensure statistical validity, allowing for consistent data, minimizing the risk of misinterpretation, and enabling meaningful comparisons between private and government sectors. As a result, several specialties were excluded from the analysis, including proctology, rheumatology, vascular surgery, hematology, and orthopedics. Ultimately, 5 types of outpatient and 6 types of inpatient facilities were included in the list of selected medical specialties. For each type of privately owned facility, the corresponding type of government-owned facility was identified to allow for parallel data extraction and comparison.

For ease of analysis, we merged the data on multidisciplinary health facilities into one category irrespective of the municipality level. Since there were more than 3 pediatric multidisciplinary hospitals, it was decided to consider them separately from the adult ones. However, there were fewer 3 children multidisciplinary outpatient clinics; therefore, their data were agglomerated with the adult outpatient facilities. Information on the types of outpatient and inpatient facilities has been available only since 2013 and, for this reason, is presented for the eight-year period from 2013 to 2020.

While analyzing visits to PHC facilities the number of visits for dental care was not accounted for. Dental visits were excluded from PHC utilization metrics due to the high variability in coding and reporting practices for dental care across regions and years. Additionally, pediatric multidisciplinary outpatient facilities were merged with adult data because fewer than three such facilities operated annually during the study period, making separate analysis statistically unreliable.

To visualize regional differences in the distribution of government and private healthcare facilities across the country, a map of Kazakhstan’s provinces as of 2020 was created.

### Calculation of healthcare indicators

Calculation of all healthcare indicators was based on recommendations of the Global Health Observatory, WHO (13). The density of outpatient and PHC facilities was calculated with the help of the following formula:


$$\begin{array}{l} \text{Health facility density}=\text{number of}\;\mathrm{all}\;\text{outpatient}\;(\mathrm{PHC})\;\\ \text{facilities in the country}\;(\text{province})/\mathrm{mid}-\text{year country}\\ \;(\text{province})\;{\text{population}}^{*}10,000. \end{array}$$

Meanwhile, the density of hospitals was calculated as follows:


$$\begin{array}{l} \text{Density of hospitals}=\text{number of hospitals of different}\;\\ \text{specializations in the country}/\mathrm{mid}-\text{year country}\;\\ {\text{population}}^{*}100,000 \end{array}$$

The density of hospitals beds was evaluated per 10,000 population:


$$\begin{array}{l} \text{Hospital}\;\mathrm{bed}\;\text{density}=\text{number of hospital beds in the}\;\\ \text{country}/\mathrm{mid}-\text{year country}\;{\text{population}}^{*}10,000 \end{array}$$

The density of PHC practitioners was calculated in accordance with the following formula:


$$\begin{array}{l} \mathrm{PHC}\;{\text{practitioners}}^{’}\;\text{density}=\text{number of}\;\mathrm{PHC}\;\text{practitioners}\;\\ \text{in the country}/\mathrm{mid}-\text{year country}\;{\text{population}}^{*}1,000 \end{array}$$

However, the number of visits to PHC facilities was calculated per capita:


$$\begin{array}{l} \text{Visits to}\;\mathrm{PHC}\;\text{facilities}=\text{number of}\;\mathrm{all}\;\text{visits to}\;\mathrm{PHC}\;\text{facilities}\;\\ \text{during}\;a\;\text{given year}/\mathrm{mid}-\text{year country population} \end{array}$$

In addition to the indicators recommended by the Global Health Observatory, we calculated a number of ratios to supplement the findings. To these belong private: government ratio, number of visits per practitioner, number of practitioners per facility, and number of hospital admissions per bed.

The linear regression analysis using log-transformed values was performed to evaluate the relative change (RC) from one parameter to another with 95% confidence intervals (95% CI). RC was derived using a log-linear regression model, where the regression coefficient (*β*) was exponentiated (e^β – 1) to calculate RC, expressed as a percentage. The dependent variable in our regression models was the annual value of each indicator (e.g., number of facilities, density, hospital admissions), while the independent variable was time (year).

## Results

**Figure 1:**
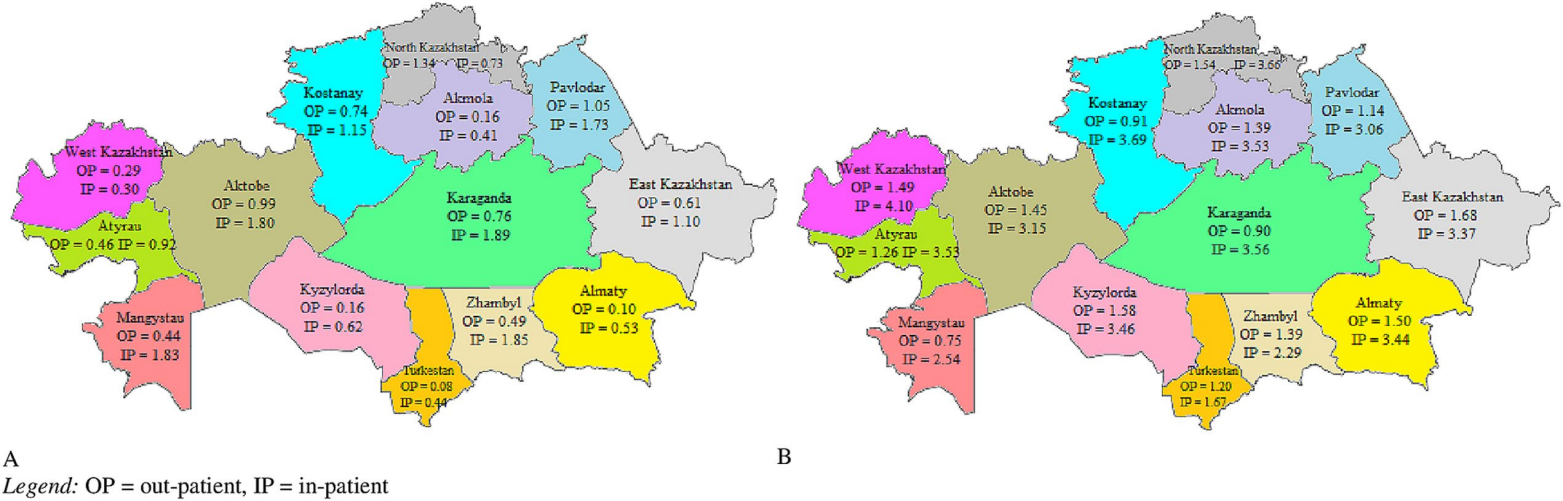
Density of government **(A)** and private **(B)** healthcare facilities per province of Kazakhstan, 2020 (QGIS version 3.22). OP = outpatient, IP = inpatient.

Table 1 reflects the density of outpatient and inpatient healthcare facilities over a period of 10 years, in relation to the type of ownership. There was a downward trend in the number of government-owned outpatient and inpatient facilities (RC constituted −3.18% and −5.30%, respectively) and this decline has become even more extensive in per capita terms. However, there was an upward trend in both the number and density of privately owned healthcare facilities and the number of private inpatient facilities increased more substantially than that of outpatient. According to Figure 1, in 2020 the distribution of outpatient and inpatient facilities was uneven across provinces of Kazakhstan. The density of healthcare facilities was the highest in 3 cities of republican significance: Nur-Sultan, Almaty, and Shymkent. Such, in 2020 the density of private outpatient and inpatient facilities in the capital city (Nur-Sultan) equaled 1.06 and 1.47, respectively, and in Almaty it constituted 1.64 and 2.36, respectively. Although the density of private outpatient facilities in Shymkent was one of the lowest in the country (0.16), the density of private inpatient facilities was the highest (2.37).

**Table 1:** Density of healthcare facilities by the type of ownership, 2011–2020.

Healthcare facilities		Year										Relative change, % (95% CI)
		2011	2012	2013	2014	2015	2016	2017	2018	2019	2020	
Government outpatient facilities	Number	2,506	2,547	2,866	2,243	2,208	2,147	2,136	2075	2026	2020	−3.18 (−4.76; −1.58)
	per 10,000 population	1.50	1.51	1.67	1.29	1.25	1.20	1.18	1.13	1.09	1.07	−4.49 (−6.05; −2.91)
Private outpatient facilities	Number (private: government ratio)	896 (1: 2.8)	912 (1: 2.79)	930 (1: 3.08)	921 (1: 2.44)	941 (1: 2.35)	979 (1: 2.19)	1,137 (1: 1.88)	1,162 (1: 1.79)	1,178 (1: 1.72)	1,149 (1: 1.76)	3.59 (2.37; 4.82)
	per 10,000 population	0.54	0.54	0.54	0.53	0.53	0.55	0.63	0.63	0.63	0.61	2.15 (0.92; 3.38)
Government inpatient facilities	Number	817	797	867	763	740	706	677	594	534	531	−5.30 (−6.75; −3.82)
	per 100,000 population	4.90	4.71	5.05	4.38	4.19	3.94	3.73	3.23	2.87	2.81	−6.59 (−8.00; −5.16)
Private inpatient facilities	Number (private: government ratio)	126 (1: 6.48)	134 (1: 5.95)	141 (1: 6.15)	148 (1: 5.16)	161 (1: 4.6)	171 (1: 4.13)	176 (1: 3.85)	194 (1: 3.06)	213 (1: 2.51)	242 (1: 2.19)	7.08 (6.20; 7.97)
	per 100,000 population	0.76	0.79	0.82	0.85	0.91	0.95	0.97	1.05	1.14	1.28	5.56 (4.66; 6.48)
Total population		16,673,077	16,909,776	17,160,774	17,417,673	17,670,579	17,918,214	18,157,337	18,395,567	18,631,779	18,879,552	1.39 (1.36; 1.43)

There was a similar trend in the number and density of PHC facilities depending on the type of ownership. Both the number and density of private PHC facilities increased (RC 5.77 and 4.93, respectively), as compared with the total number and density of PHC facilities in the country (RC −0.75 and −2.13, respectively). Likewise, there was an increase in the number of visits to private PHC facilities (RC was 9.28% or 7.72% in per capita terms). Although there was a growth in the total number and density of PHC practitioners, those of private practitioners increased more considerably. In general, private PHC facilities tended to be larger than the average PHC facility. For example, in 2020 the number of private practitioners per facility was almost 2 times higher than that of the average PHC facility (23.90 vs. 13.11). Of interest is the fact that practitioners working in the private PHC had lower workload during the entire period of observation, which is expressed as the number of visits per practitioner (Table 2).

**Table 2:** Characteristics of primary healthcare (PHC) facilities by the type of ownership, 2011–2020.

PHC indicators		Year										Relative change, % (95% CI)
		2011	2012	2013	2014	2015	2016	2017	2018	2019	2020	
Number of PHC facilities	Total	1961	1934	1908	1870	1849	1830	1836	1844	1828	1833	−0.75 (−1.05; −0.44)
	Per 10,000 population	1.18	1.14	1.11	1.07	1.05	1.02	1.01	1.00	0.98	0.97	−2.13 (−2.46; −1.79)
	Private (private: total ratio)	230 (1: 8.53)	160 (1: 12.09)	91 (1: 20.97)	69 (1: 27.1)	78 (1: 23.71)	107 (1: 17.1)	142 (1: 12.93)	179 (1: 10.3)	236 (1: 7.75)	248 (1: 7.39)	5.77 (−6.12; 19.17)
	Per 10,000 population	0.14	0.09	0.05	0.04	0.04	0.06	0.08	0.10	0.13	0.13	4.93 (−8.79; 20.70)
Visits to PHC facilities	Total visits	84,053,870	81,961,285	79,868,700	83,253,500	78,925,000	81,253,600	81,876,600	76,819,400	72,583,200	79,821,500	−0.92 (−1.79; −0.05)
	Per capita	5.04	4.85	4.65	4.78	4.47	4.53	4.51	4.18	3.90	4.23	−2.27 (−3.12; −1.42)
	Visits to private (private: total ratio)	7,126,360 (1: 11.79)	6,447,030 (1: 12.71)	5,767,700 (1: 13.85)	5,231,800 (1: 15.91)	6,182,800 (1: 12.77)	7,023,300 (1: 11.57)	8,327,500 (1: 9.83)	10,494,100 (1: 7.32)	12,930,200 (1: 5.61)	12,774,100 (1: 6.25)	9.28 (4.02; 14.80)
	Per capita	0.43	0.38	0.34	0.30	0.35	0.39	0.46	0.57	0.69	0.68	7.72 (2.50; 13.21)
PHC practitioners	Total number	18,377	18,547	18,718	19,480	19,390	20,548	21,151	21,825	23,467	24,039	3.16 (2.54; 3.78)
	Per 1,000 population	1.10	1.10	1.09	1.12	1.10	1.15	1.16	1.19	1.26	1.27	1.73 (1.12; 2.35)
	Visits per practitioner	4573.86	4419.11	4266.95	4273.79	4070.40	3954.33	3871.05	3519.79	3092.99	3320.50	−3.96 (−5.01; −2.90)
	Practitioners per facility	9.37	9.59	9.81	10.42	10.49	11.23	11.52	11.84	12.84	13.11	3.94 (3.50; 4.98)
	Number of private (private: total ratio)	3,141 (1: 5.85)	2,312 (1: 8.02)	1,484 (1: 12.61)	1,485 (1: 13.12)	1859 (1: 10.43)	2,183 (1: 9.41)	2,973 (1: 7.11)	4,356 (1: 5.01)	5,533 (1: 4.24)	5,927 (1: 4.06)	12.51 (2.13; 23.95)
	Per 1,000 population	0.19	0.14	0.09	0.09	0.11	0.12	0.16	0.24	0.30	0.31	10.49 (0.53; 21.44)
	Visits per private practitioner	2268.82	2788.51	3886.60	3523.10	3325.88	3217.27	2801.04	2409.11	2336.92	2155.24	−2.87 (−7.58; 2.08)
	Private practitioners per facility	13.66	14.45	16.31	21.52	23.83	20.40	20.94	24.34	23.44	23.90	6.37 (3.22; 9.61)

Tables 3, 4 present the analysis of selected outpatient and inpatient facilities by the type of ownership for the period from 2013 to 2020. As shown in the tables, there is uneven presence of the private healthcare sector in different medical specialties. Dentistry, ophthalmology, maternity, multidisciplinary clinics, and health resorts were the commonest types of outpatient facilities, while narcology, ophthalmology, maternity, multidisciplinary hospitals along with palliative and nursing care facilities were the most frequent types of hospitals. Overall, both the number and density of all selected government-owned outpatient and inpatient facilities have been on a downward trend. The only exception was palliative and nursing care, which showed an upward trend over the past 8 years (RC was 10.87% or 12.02% in per capita terms). As for the private healthcare sector, the number and density of ophthalmology, narcology, multidisciplinary, as well as nursing and palliative care facilities increased over time, while those of dentistry and maternity facilities as well as health resorts were subjected to decline. As of 2020, there was a substantial predominance of privately owned facilities over government-owned facilities in such medical fields as palliative and nursing care, ophthalmology, dentistry, promotion of healthy lifestyle, and multidisciplinary healthcare.

**Table 3:** Characteristics of selected outpatient facilities by the type of ownership, 2013–2020.

Outpatient facilities		Year								Relative change, % (95% CI)
		2013	2014	2015	2016	2017	2018	2019	2020	
Government	Number of maternity clinics	24	23	21	24	27	14	5	4	−22.37 (−35.07; −7.18)
	Per 10,000 population	0.014	0.013	0.012	0.013	0.015	0.008	0.003	0.002	−23.07 (−35.62; −8.07)
	Number of ophthalmology clinics	4	4	4	4	4	3	2	2	−10.35 (−16.20; −4.10)
	Per 10,000 population	0.002	0.002	0.002	0.002	0.002	0.002	0.001	0.001	−9.43 (−16.86; −1.33)
	Number of dentistry clinics	16	16	15	15	13	10	11	9	−8.28 (−11.21; −5.26)
	Per 10,000 population	0.009	0.009	0.008	0.008	0.007	0.005	0.006	0.005	−8.74 (−12.13; −5.23)
	Number of multidisciplinary clinics	482	466	475	474	469	454	415	409	−2.21 (−3.48; −0.92)
	Per 10,000 population	0.281	0.268	0.269	0.265	0.258	0.247	0.223	0.217	−3.52 (−4.73; −2.30)
	Number of health resorts	37	37	37	35	36	34	25	15	−9.63 (−16.73; −1.92)
	Per 10,000 population	0.022	0.021	0.021	0.020	0.020	0.018	0.013	0.008	−11.16 (−17.92; −3.85)
Private	Number of maternity clinics (private: government ratio)	7 (1: 3.43)	5 (1: 4.6)	5 (1: 4.2)	5 (1: 4.8)	6 (1: 4.5)	7 (1: 2)	3 (1: 1.67)	3 (1: 1.33)	−8.32 (−17.43; 1.80)
	Per 10,000 population	0.004	0.003	0.003	0.003	0.003	0.004	0.002	0.002	−6.91 (−14.10; 0.88)
	Number of ophthalmology clinics (private: government ratio)	3 (1: 1.33)	3 (1: 1.33)	4 (1: 1)	4 (1: 1)	5 (1: 0.8)	7 (1: 0.43)	7 (1: 0.29)	7 (1: 0.29)	15.45 (10.91; 20.18)
	Per 10,000 population	0.002	0.002	0.002	0.002	0.003	0.004	0.004	0.004	13.72 (7.37; 20.46)
	Number of dentistry clinics (private: government ratio)	311 (1: 0.05)	290 (1: 0.06)	289 (1: 0.05)	304 (1: 0.05)	308 (1: 0.04)	302 (1: 0.03)	292 (1: 0.04)	303 (1: 0.03)	0.00 (−1.15; 1.15)
	Per 10,000 population	0.181	0.166	0.164	0.170	0.170	0.164	0.157	0.160	−1.70 (−3.13; −0.26)
	Number of multidisciplinary clinics (private: government ratio)	505 (1: 0.95)	607 (1: 0.77)	626 (1: 0.76)	648 (1: 0.73)	799 (1: 0.59)	817 (1: 0.56)	839 (1: 0.49)	801 (1: 0.51)	7.22 (4.25; 10.28)
	Per 10,000 population	0.294	0.348	0.354	0.362	0.440	0.444	0.450	0.424	5.78 (2.87; 8.78)
	Number of health resorts (private: government ratio)	42 (1: 0.88)	39 (1: 0.95)	39 (1: 0.95)	42 (1: 0.83)	30 (1: 1.2)	15 (1: 2.27)	23 (1: 1.09)	14 (1: 1.07)	−14.88 (−22.51; −6.50)
	Per 10,000 population	0.024	0.022	0.022	0.023	0.017	0.008	0.012	0.007	−18.61 (−44.99; 20.43)

**Table 4:** Characteristics of selected inpatient facilities by the type of ownership, 2013–2020.

Inpatient facilities		Year								Relative change, % (95% CI)
		2013	2014	2015	2016	2017	2018	2019	2020	
Government	Number of maternity hospitals	44	45	44	41	40	35	24	22	−9.85 (−14.42; −5.02)
	Per 100,000 population	0.26	0.26	0.25	0.23	0.22	0.19	0.13	0.12	−10.95 (−15.19; −6.51)
	Number of ophthalmology hospitals	4	4	4	4	4	3	2	2	−10.35 (−16.20; −4.10)
	Per 100,000 population	0.02	0.02	0.02	0.02	0.02	0.02	0.01	0.01	−9.43 (−16.86; −1.33)
	Number of narcology hospitals	9	6	4	4	3	3	3	3	−13.63 (−20.05; −6.69)
	Per 100,000 population	0.05	0.03	0.02	0.02	0.02	0.02	0.02	0.02	−9.56 (−17.57; −0.78)
	Number of adult multidisciplinary hospitals	261	256	262	261	254	254	258	259	−0.16 (−0.55; 0.23)
	Per 100,000 population	1.52	1.47	1.48	1.46	1.40	1.38	1.38	1.37	−1.53 (−1.99; −1.07)
	Number of pediatric multidisciplinary hospitals	30	29	28	28	27	24	23	22	−4.46 (−5.62; −3.28)
	Per 100,000 population	0.17	0.17	0.16	0.16	0.15	0.13	0.12	0.12	−5.63 (−7.29; −3.94)
	Number of palliative and nursing care facilities	2	2	3	3	4	6	4	3	10.87 (−0.59; 23.66)
	Per 100,000 population	0.01	0.01	0.02	0.02	0.02	0.03	0.02	0.02	12.02 (0.85; 24.42)
Private	Number of maternity hospitals (private: government ratio)	7 (1: 6.29)	5 (1: 9)	5 (1: 8.8)	5 (1: 8.2)	6 (1: 6.67)	7 (1: 5)	5 (1: 4.8)	4 (1: 5.5)	−3.19 (−9.87; 3.98)
	Per 100,000 population	0.04	0.03	0.03	0.03	0.03	0.04	0.03	0.02	−4.64 (−11.46; 2.70)
	Number of ophthalmology hospitals (private: government ratio)	3 (1: 1.33)	3 (1: 1.33)	4 (1: 1)	4 (1: 1)	5 (1: 0.8)	7 (1: 0.43)	11 (1: 0.18)	11 (1: 0.18)	23.15 (16.45; 30.24)
	Per 100,000 population	0.02	0.02	0.02	0.02	0.03	0.04	0.06	0.06	20.51 (11.75; 29.95)
	Number of narcology hospitals (private: government ratio)	3 (1: 3)	6 (1: 1)	8 (1: 0.5)	9 (1: 0.44)	8 (1: 0.38)	7 (1: 0.43)	7 (1: 0.43)	6 (1: 0.5)	6.27 (−6.23; 20.42)
	Per 100,000 population	0.02	0.03	0.05	0.05	0.04	0.04	0.04	0.03	4.11 (−7.54; 17.23)
	Number of adult multidisciplinary hospitals (private: government ratio)	111 (1: 2.35)	120 (1: 2.13)	132 (1: 1.98)	142 (1: 1.84)	145 (1: 1.75)	163 (1: 1.56)	170 (1: 1.52)	196 (1: 1.32)	7.89 (6.79; 8.99)
	Per 100,000 population	0.65	0.69	0.75	0.79	0.80	0.89	0.91	1.04	6.39 (5.27; 7.51)
	Number of pediatric multidisciplinary hospitals (private: government ratio)	4 (1: 7.5)	1 (1: 29)	1 (1: 28)	1 (1: 28)	1 (1: 27)	1 (1: 24)	2 (1: 11.5)	2 (1: 11)	−1.64 (−20.51; 21.72)
	Per 100,000 population	0.02	0.01	0.01	0.01	0.01	0.01	0.01	0.01	−5.61 (−13.01; 2.41)
	Number of palliative and nursing care facilities (private: government ratio)	7 (1: 0.29)	7 (1: 0.29)	6 (1: 0.5)	6 (1: 0.5)	7 (1: 0.57)	7 (1: 0.86)	8 (1: 0.5)	9 (1: 0.33)	3.69 (−0.53; 8.09)
	Per 100,000 population	0.04	0.04	0.03	0.03	0.04	0.04	0.04	0.05	3.28 (−2.82; 9.77)

The number and density of beds in government-owned hospitals experienced a decline, while those in privately owned facilities increased over time. In per capita terms, there was a decline in the rate of admissions to government hospitals (RC was −1.12%) and a growth in the number of admissions to private hospitals (RC = 5.45%). Considering the number of admissions per bed, there was a growing trend in the government-owned hospitals and a declining trend in the privately owned hospitals (RC 1.31% vs. −1.73%) as shown in Table 5.

**Table 5:** Hospital beds and hospital admissions by the type of ownership, 2011–2020.

Hospital beds and admissions		Year										Relative change, % (95% CI)
		2011	2012	2013	2014	2015	2016	2017	2018	2019	2020	
Hospital beds	Government	111,455	111,456	110,737	98,566	95,772	92,606	91,734	89,848	88,722	112,885	−1.67 (−3.92; 0.63)
	Per 10,000 population	66.85	65.91	64.53	56.59	54.20	51.68	50.52	48.84	47.62	59.79	−3.02 (−5.26; 0.73)
	Private (private: government ratio)	6,200 (1: 17.98)	6,199(1: 17.98)	6,461(1: 17.14)	6,653(1: 14.82)	6,717(1: 14.26)	7,473(1: 12.39)	7,731(1: 11.87)	8,523(1: 10.54)	9,810(1: 9.04)	12,735(1: 8.86)	7.31 (4.66; 10.01)
	Per 10,000 population	3.72	3.67	3.76	3.82	3.80	4.17	4.26	4.63	5.27	6.75	5.83 (3.19; 8.54)
Hospital admissions	Government	2,613,200	2,663,160	2,605,119	2,540,971	2,550,816	2,652,702	2,688,715	2,681,567	2,683,834	2,618,606	0.26 (−0.25; 0.77)
	Per 10,000 population	1567.32	1574.92	1518.07	1458.85	1443.54	1480.45	1480.79	1457.72	1440.46	1387.01	−1.12 (−1.63; −0.60)
	Admissions per bed	22.21	22.64	22.23	24.15	24.89	26.51	27.03	27.26	27.24	20.85	1.31 (−1.17; 3.85)
	Private (private: government ratio)	168,300(1: 15.53)	178,572(1: 14.91)	178,904(1: 14.56)	189,930(1: 13.38)	182,532(1: 13.97)	197,902(1: 13.40)	214,738(1: 12.52)	245,044(1: 10.94)	256,334(1:10.47)	268,332(1: 9.76)	5.45 (4.07; 6.84)
	Per 10,000 population	100.94	105.60	104.25	109.04	103.30	110.45	118.27	133.21	137.58	142.13	4.00 (2.62; 5.40)
	Admissions per bed	27.15	28.81	27.69	28.55	27.17	26.48	27.78	28.75	26.13	21.07	−1.73 (−3.69; 0.26)

## Discussion

To the best of our knowledge, this is the first study exploring transformation of the private health sector in the Republic of Kazakhstan following a series of healthcare plans. In contrast with the government-owned health facilities, the overall number of privately owned facilities increased over time, although this growth was less obvious in per capita terms. The number and density of privately owned PHC facilities grew more substantially than those of privately owned outpatient facilities, but in absolute terms the number of outpatient facilities was significantly higher. In other words, by 2020 there were just 1.76 government-owned outpatient facilities per each privately owned facility and in case with PHC facilities this rate constituted 1: 7.39. Besides, there is uneven presence of private health sector in different medical specialties with maternity, ophthalmology, dentistry, narcology, multidisciplinary, as well as palliative and nursing care being the most common. Several interrelated reasons can explain the regional disparity in private healthcare presence. Firstly, large cities such as Almaty, Nur-Sultan, and Shymkent attract more private investments due to their higher population density, better infrastructure, and greater concentration of solvent demand. These urban centers also offer better availability of trained healthcare personnel, access to advanced technologies, and proximity to regulatory bodies, all of which lower the operational risks for private investors. In contrast, rural and remote regions face infrastructural limitations, shortages of qualified staff, and reduced purchasing power, which diminish the financial attractiveness for private operators. Secondly, regional disparities may reflect differences in local governance practices, including how regional health departments engage with and support private providers. Licensing procedures, reimbursement mechanisms, and access to government contracts through the State Guaranteed Benefits Package (SGHBP) may be more efficiently administered in urban provinces. Lastly, the uneven distribution may stem from historical development patterns and urbanization processes, which were inherited from the Soviet period and still shape the healthcare infrastructure landscape in Kazakhstan today. PHC practitioners employed by the government-owned facilities admit more patients than practitioners of the private PHC facilities do.

As a legacy of the Soviet Union, Kazakhstan possesses a broad net of healthcare facilities, having one of the highest densities in the world. Correspondingly, the hospital beds density in Kazakhstan is comparable to that in other post-soviet economies and some high-income countries. As of 2017, the density of hospital beds in Bulgaria was 74.54 per 10,000 population, in Hungary and Romania it was 70.2 and 68.92, respectively, while in Russian Federation there were 80.5 beds per 10,000 population (14). Globally, many countries decreased the hospital beds density over time due to adherence to hospital avoidance strategies and Kazakhstan was no exception (15). However, in 2020 Kazakhstan faced the urgent need for rapid deployment of additional hospital beds due to the escalation of the COVID-19 pandemic, which became a major public health crisis (16). The pandemic had a profound, albeit uneven, impact on the healthcare system, including the private sector. In response to the emergency, the government mobilized both public and private healthcare providers to expand inpatient capacity. Although private hospitals were not originally intended for large-scale infectious disease management, many adjusted quickly by reallocating resources, setting up COVID-19 units, and entering into short-term service contracts under the State Guaranteed Benefits Package. This led to a temporary increase in the visibility and involvement of private providers in inpatient care. At the same time, however, private outpatient and diagnostic clinics experienced considerable disruptions due to lockdown measures, decreased patient flow, and financial constraints. As a result, the pandemic exposed both the potential and the limitations of the private health sector in addressing large-scale public health emergencies. These dynamics likely contributed to the fluctuations in facility numbers and utilization rates observed in 2020, highlighting the need for more resilient and better-integrated public-private collaboration mechanisms in future crises.

Non-profit (NP) healthcare organizations are very common and in some countries they deliver the bulk of health services. It is noteworthy that established market economies often have a larger share of NP health sector as compared with the emerging or transition economies (17). For example, in the Netherlands hospitals are NP organizations and are not allowed to generate income; in Australia residential care is mostly provided by NP organizations and in France two thirds of hospital beds belong to government-owned or NP facilities (18). Moreover, in Canada 80% of people prefer NP model of care over the private for-profit care and 53% select a mixed model incorporating both types of providers (19). However, as is the case in many transition countries, the NP health sector in Kazakhstan is significantly underrepresented, with the vast majority of private healthcare facilities operating on a for-profit basis.

Several explanations could account for why NP health sector is lacking in post-soviet countries. Firstly, this is due to a continuing influence of state socialism, which manifests in strong centralization of social and political power. As a result, civil society institutions are underdeveloped and the public is short of capacity for self-organization. Secondly, there is a historical absence of any forms of working relationship between government authorities and NP sector. Thirdly, the legal frameworks for private NP activity are not sufficiently developed. Furthermore, the relatively small urban middle class, that is often a driver of social division might limit domestic support for NFP initiatives (17). In Kazakhstan, the scarcity of NFP health sector likely contributes to high out-of-pocket healthcare spending, which reached 38.5% in 2018. Alarmingly, some population groups spend up to 33% of their income on medical care, posing a significant threat to their financial stability (20).

In Kazakhstan the share of private healthcare providers increases year after year. Such, in 2014 27.4% of medical services provided within the state guaranteed health benefits package (SGHBP) were delivered by the privately owned facilities and in 2018 this proportion constituted 43% (7). It is noteworthy to mention that the SGHBP is financed from the state budget and thus, the government becomes an important payer for medical services delivered by the private health sector. Public-private partnership schemes are widely used in Kazakhstan to compensate for health expenditures, but imperfection of legislative framework poses additional barriers. However, the MoH makes many efforts to eliminate such barriers via a systematic supplementation and amendment of regulatory documents. While implementing the public-private partnership mechanisms in the healthcare sector it is important to bear in mind that long payback period is common and thus, one should not expect a quick success (21).

It is not quite obvious why in Kazakhstan the private health sector prefers to operate in certain medical fields. Dental enterprises became very common from the early years of independence and by far remain the most common type of private clinic. The drop in number of narcology and alcoholism clinics, both government- and privately owned, is due to decreasing alcohol consumption, which is observed in the majority of post-soviet countries (22). The reason why ophthalmology clinics gained much popularity is not clear, but might be due to the fact that eye care is relatively inexpensive and is well-adjusted to the purchasing power of most citizens. Palliative and nursing care facilities were literally non-existent before and therefore, are strongly needed. Nevertheless, private multidisciplinary medical centers have proven convenient and are well regarded by the public.

Kazakhstan’s private healthcare growth reflects post-Soviet transitions. Inheriting a centralized Semashko system, the country has expanded private services in some areas, yet progress is limited by weak regulation and a lack of non-profit providers. Addressing these legacy issues is key to building a more balanced and effective health system (23).

The present study has certain limitations secondary to its retrospective cross-sectional design and dependence on pre-collected data, which prevented more detailed disaggregation impossible. Although there is often a significant overlap between public and private health sectors as they tend to share professionals and infrastructure, we were able to track such information as the statistical yearbooks enable sufficient differentiation. While all healthcare facilities, regardless of ownership, are legally required to report to provincial health authorities, we acknowledge that reporting completeness and data quality may vary between public and private facilities due to differences in internal systems or motivations. In general, such data are needed for decision-makers to tailor public health strategies targeted on the stewardship of private health sector that would help to improve availability and affordability of medical services.

Further Research Implications. Future research will focus on assessing the quality and efficiency of services in private and public healthcare institutions, exploring the barriers to the development of the non-profit sector, and evaluating the impact of public-private partnerships on equity and access to healthcare in Kazakhstan.

## Data Availability

The original contributions presented in the study are included in the article/supplementary material, further inquiries can be directed to the corresponding author.
